# The impact of foreign direct investment on green innovation efficiency: Evidence from Chinese provinces

**DOI:** 10.1371/journal.pone.0298455

**Published:** 2024-02-14

**Authors:** Shen Zhong, Zhicheng Zhou, Hongjun Jing

**Affiliations:** 1 School of Finance, Harbin University of Commerce, Harbin, China; 2 School of Public Finance and Administration, Harbin University of Commerce, Harbin, China; University of Sargodha, PAKISTAN

## Abstract

Improving green innovation efficiency (GIE) is the key to achieve high-quality economic development in China, and the introduction of foreign direct investment (FDI) has become an important path choice to promote the GIE. Based on the data of 30 provinces in China, this paper explores the linear and nonlinear effects of FDI on GIE from both quantity and quality perspectives, and further analyzes the mediating role of environmental regulation level. The results show that: (1) From 2011 to 2020, the GIE of all provinces in China generally shows an upward trend. (2) The quantity and quality of FDI have a significant positive impact on the improvement of GIE in China’s provinces, and this impact has regional heterogeneity. (3) The quantity and quality of FDI can promote the improvement of GIE in China through the level of environmental regulation (ER). (4) With the level of knowledge accumulation and GIE as the threshold variables, the quantity and quality of FDI have a single threshold effect on the GIE of China’s provinces. The conclusions of this study provide some policy implications for local governments to make full use of FDI to perform green innovation activities.

## 1. Introduction

In the past 30 years, the global environment has not only been polluted and destroyed but also faced a severe crisis, which poses a significant threat to the survival and development of humanity. With the rapid growth of the global economy, the solution to environmental problems is essential for the sustainable development of human beings. Academics have verified this view. For example, Xue et al (2019) proposed that reducing air pollution and greenhouse gas emissions can improve people’s health [1]. To address environmental concerns, 197 countries agreed to the Paris Climate Agreement, which aims to substantially lower global greenhouse gas emissions and limit the global temperature rise to 2 degrees Celsius this century. Simultaneously, China has made noteworthy economic progress with a focus on economic development. According to the World Bank (1992–2022), China’s gross domestic product (GDP) has increased significantly, from $0.43 trillion in 1992 to $17.82 trillion in 2022. Nevertheless, the substantial economic growth model has caused severe environmental pollution issues in China, notably air pollution, which negatively impacts air quality, human health, and climate [2]. According to data from the World Bank (1992–2022), China’s carbon emissions for 2020 are projected to reach nearly 11 billion tonnes, a significant increase from 2.4 billion tonnes in 1992, with an average annual growth rate of 5.5%. As such, it is imperative to address the issue of measuring the relationship between economic growth and environmental protection in China. Green innovation can help reduce environmental pollution by providing green technologies, products, and processes to Chinese provinces. This can lead to economic benefits and promote coordinated development of the economy and environment [3]. In this context, promoting green innovation is crucial to reconcile economic growth with environmental protection and to drive high-quality development in China’s economy. Scholars have introduced the GIE index to gauge green innovation development [4, 5]. This metric measures the extent of green innovation development in a region by calculating the input-output ratio of innovation resources and environmental externalities.

Improving the GIE has become crucial for local authorities and academics. The Chinese Government has long prioritized FDI, welcoming it through the Regulations on Encouraging Foreign Direct Investment 1986. Since then, FDI has increased in a significant manner in China. According to the China Foreign Investment Development Report (2022), China utilized FDI worth 111.72 billion US dollars in 2012, which increased to 173.48 billion US dollars in 2021, representing a 55% increase. China ranks second in the world in terms of annual FDI inflows. Large-scale FDI has been instrumental in the rapid growth of China’s economy [6, 7]. During the initial phases of China’s growth, FDI predominantly targeted manufacturing and real estate industries. According to China’s statistical yearbook 1998, FDI in China’s real estate and manufacturing industries amassed over 37 billion US dollars, accounting for 72% of the year’s FDI. By promoting China’s development, FDI has caused greater damage to China’s environment. As China’s economy has developed, its industrial focus has shifted towards the service industry, with FDI gradually being directed towards these sectors. According to China’s statistical yearbook, the industries that received FDI in 2021 have undergone significant changes compared to 1998. Notably, the service industry accounted for approximately 43.8% of all FDI in the country throughout the year, while the proportion of the real estate and manufacturing sectors decreased to 33.1%. Against industrial structural upgrades, FDI can facilitate green innovation and mitigate environmental pollution through technology spillover and independent innovation. This, in turn, profoundly impacts the GIE across China’s provinces.

This study addresses the research question of how FDI impacts the GIE in China’s provinces. To achieve this, panel data from 30 Chinese regions and cities between 2011 and 2020 were used to systematically investigate the effect of FDI quantity and quality on each province’s GIE from the perspectives of absorptive capacity theory, competitive cooperation theory and local government theory. The study finds that both the quantity and quality of FDI significantly impact GIE in every region, even after controlling for endogenous issues and conducting robustness testing. Moreover, FDI can help improve environmental regulation, promoting GIE. The analysis of regional heterogeneity reveals notable differences in the effect of FDI quantity and quality on GIE across regions. As the local knowledge accumulation level improves, the impact of both the quantity and quality of FDI on GIE shifts from negative to positive. The subsequent sections of this paper are structured as follows: the second part consists of a literature review, the third part explains the theoretical framework and research assumptions, the fourth part describes the model setting and data, the fifth part details the empirical analysis and the final section summarises the empirical results and includes appropriate policy recommendations.

## 2. Literature review

The OECD defines green innovation as an innovative activity that consciously or unconsciously makes technological progress and improves environmental efficiency. This helps conserve production and living resources through technological spillover and environmental externalities during innovation. The GIE quantitatively describes the level of development of regional green innovation based on the input-output ratio of resources and environmental impact [4, 5]. In GIE research, scholars concentrate on measuring and analyzing GIE and its influencing factors. To measure GIE, parametric and non-parametric methods are used, with stochastic frontier analysis (SFA) being the representative parametric method [8, 9]; this method uses econometric models to measure the growth structure [10, 11]; Data Envelopment Analysis (DEA) is a method that evaluates the effectiveness or benefit of a decision-making unit (DMU) about multi-input and multi-output indicators using linear programming techniques and based on relative efficiency [12, 13]. The amount of literature using the stochastic frontier analysis method is comparatively low compared to the existing literature. This is due to certain defects in the traditional SFA model [14], while the DEA method is better suited to fit multi-output production activities with unexpected outputs and can prevent the strong bias of SFA towards the model form setting and random interference normal distribution. Therefore, it is commonly applied to assess efficiency and productivity under environmental restrictions [15–17]. There are mainly two models in the literature of using DEA to measure the GIE: EBM model and SBM model, in which EBM model is a mixed model including radial and SBM distances [18]. Xu et al. (2020) used the Super-SBM model to measure the GIE of cities in the Yangtze River Economic Zone [19]. Wang et al. (2022) evaluated the GIE (GIE) of 285 cities in China using the EBM-DEA model [20]. Wang et al. (2023) used the GSE-EBM model to measure the GIE of 285 cities in China [21]. The results showed that the GIE of Chinese cities showed a fluctuating upward trend, with pronounced regional differences. The regional differences gradually decreased from the eastern coastal areas to the central, western, and northeastern regions. Zhuang et al. (2022) used the EBM model to measure the GIE of 30 provinces in China [22]. Miao et al (2021) constructed a two-stage SBM-DEA model including energy and non-performing output to measure the GIE, and the results show that the GIE in eastern China has been higher than that in other regions [4].

Scholars study the influencing factors of GIE mainly from environmental regulation, government support, industrial structure, and so on. The existing literature has studied the impact of environmental regulation on the GIE from various perspectives. Fang et al (2020) argue that environmental regulation significantly negatively impacts the GIE in heavy pollution industries [23]. Zhang et al (2020) discussed the impact of Xi’an environmental regulation on its GIE. They found that market-based environmental regulation is more effective than command-and-control environmental regulation, and there is an inverted U-shaped relationship between environmental regulation and GIE [24]. Li & Du (2021) found that environmental law has a significant spatial spillover effect on the GIE of Chinese cities, showing a U-shaped result of first inhibition and then promotion [25]. Fan et al (2021) argue that there is a positive U-sharped relationship between environmental regulation and GIE and supports Porter’s hypothesis at the scale of Chinese cities [26]. Xu et al (2022) believe that ER significantly positively affects GIE only when the environmental regulation range is between 0.595 and 0.899 in the Yellow River Basin [27]. To sum up, due to the differences in research objects, scholars lack consistent conclusions about the impact of ER on GIE but generally believe that the effects of environmental regulation on GIE exist. Local government’s emphasis on green innovation can effectively promote the GIE in the stage of knowledge absorption and commercialization [28]. Liu et al (2022) argue that local government competition will lead to a mismatch between innovative talents and innovative capital, thus reducing GIE [29]. Li & Zeng (2020) believe that there is an inverted "U" relationship between government R&D support and the GIE of IP institutions [30]. Yi et al (2020) discussed the relationship between government R&D subsidies and the GIE of the Yangtze River Economic Zone, and the results showed that government R&D subsidies are conducive to improving the GIE of the Yangtze River Economic Zone [31]. From the above, local governments can effectively influence the change of GIE in the region. Industrial structure promotes GIE in the Pearl River Delta region [32]. Li (2023) intensely studied the impact of industrial structure on the GIE of 30 provinces and cities in China and found that the rationalization of industrial design has an inhibitory effect on the GIE of the region and its surrounding areas and the upgrading of industrial structure has a promoting effect on the GIE of the region and its surrounding areas [33]. In summary, DEA is commonly used among scholars to measure GIE, specifically SBM and EBM. However, DDF is not frequently utilized. This model provides decision makers with the option to adjust weights and directions, allowing for greater flexibility and control. Consequently, the model can better assess GIE. Nonetheless, this approach does not account for the influences of FDI on GIE, which are significant and should not be overlooked in exploratory analyses.

According to the Organization for Economic Cooperation and Development (OECD) definition, direct investment refers to acquiring individuals or enterprises living in one economy to enterprises in another through cross-border investment. It aims to establish a long-term interest relationship. Direct investment can be divided into outward FDI (OFDI) and inward FDI (IFDI). In this paper, FDI refers to inward FDI. Scholars mainly focus on the following two aspects: whether the quantity (FDI_1_) and quality (FDI_2_) of FDI can promote the GIE and the research methods. The existing literature has different opinions on whether FDI_1_ or FDI_2_ can boost the GIE and has the following views. The first view is that FDI_1_ or FDI_2_ positively impacts the GIE. Luo et al (2021) argue that the FDI_1_ plays a positive role in green innovation in developing countries, which verifies the "pollution halo hypothesis" [34]. Xu et al (2021) believe that the FDI_1_ can effectively promote the improvement of GIE in China’s provinces [35]. Chen et al (2023) believe that the FDI_1_ significantly impacts GIE in the eastern and western regions of China. Still, there is no significant negative impact on the GIE in the central area [36]. Feng et al (2018) argue that FDI_2_ positively impacts the GIE of China’s manufacturing industry [37]. The second view is that FDI_1_ or FDI_2_ hurts the GIE. Jiang et al (2020)analyzed the panel data of 211 cities and found that improving FDI_2_ can inhibit the GIE of Chinese cities [38]. Xu & Li (2021) hold that the FDI_2_ has a significant negative impact on the green innovation capability of developing countries [35] A threshold effect exists between FDI_2_ based on innovation activities and GIE. When the innovation activities are below the threshold, the FDI_2_ hurts green innovation, and vice versa. In the study of 36 cities in the Yangtze River Delta, Zeng et al (2021) found that the quality of FDI is a negative determinant of the co-integration of GIE in the Yangtze River Delta region [39]. The third view is that the impact of FDI_1_ or FDI_2_ on GIE needs to be revised. Song & Han (2022) believes that the effect of FDI_1_ on GIE is "U" shaped, and the driving impact of FDI_1_ on GIE in the western region is more significant than that in the central and eastern areas [40]. Qin et al (2022) believe that the FDI_1_ can significantly promote green innovation in Chinese cities, and the promotion effect of FDI on green innovation has nonlinear characteristics; that is, it is meaningful only when the absorptive capacity exceeds the threshold [41]. To sum up, due to the differences in research objects, research data, and research ideas, scholars need consistent conclusions about the impact of the FDI_1_ or FDI_2_ on the GIE but generally believe that this impact exists. From the perspective of research methods, scholars have used various empirical methods to demonstrate this impact. Specific practical methods include the SYS-GMM model [34], the geographically weighted regression (GWR) model [38], the threshold model [35, 41], the two-layer stochastic boundary model [40], and the spatial Durbin model (SDM) [39], there are also mechanism analysis, Such as the mediating effect model [38] and the moderating effect model [36]. The literature has explored the relationship between FDI and GIE, but studies have predominantly examined either the quantity or the quality of FDI, failing to consider both aspects.

To sum up, the existing literature research has the following deficiencies: (1) In terms of research perspective, the existing research only analyzes FDI from the standpoint of quantity or quality and needs more analysis from more perspectives. (2) Regarding research theory, some studies have proved that environmental regulation has an impact on GIE, and FDI has an impact on environmental regulation. They still need to verify that the level of environmental regulation has a mediating effect. (3) Regarding research methods, the existing studies have yet to systematically and deeply analyze whether the mechanism of FDI on GIE is affected by the level of knowledge and technology in China’s provinces. In this regard, this paper has the following innovations: (1) Innovation of research perspective. This paper analyzes the relationship between FDI and GIE from the quantity and quality perspective and makes an empirical analysis. (2) Innovation of research theory. This paper demonstrates that FDI can affect GIE through environmental regulation and empirically tests the mediating effect of environmental regulation by using the mediating effect model, which enriches the theoretical analysis system of the relationship between FDI and GIE. (3) Innovative application of methods. In this paper, the super-efficiency DDF-GML model is used to measure the GIE of Chinese provinces, and the threshold model is used to discuss the nonlinear impact of FDI on GIE. Considering that the endogeneity of the model may lead to bias, SYS-GMM and 2SLS model are used for further estimation.

## 3. Theoretical framework and research hypotheses

Based on the absorptive capacity theory, the theory of competitive cooperation among firms, and the theory of local government competition, this paper conducts a detailed analysis of the relationship between FDI and GIE. The theory of absorptive capacity was put forward by Cohen and Levinthal in 1990. Its central concept is that organizational innovation largely depends on the ability to recognize the importance of new external knowledge, assimilate it and apply it for commercial goals, i.e. the absorptive capacity of the organization. This capacity is largely influenced by the previous knowledge level of the organization [42]. Nalebuff and Brandenburger developed the theory of competitive cooperation between firms in 1996 [43, 44], The central proposition posits that conducting business is a unique type of game, one that is non-zero-sum and capable of producing mutually beneficial outcomes. Consequently, competition can have a constructive influence on firms. The theory of municipal government competition, advanced by Breton in 1998, holds that governments are inherently competitive and exhibit this trait in how they allocate resources and control, as well as when competing to provide public goods and services [45].

### 3.1 The impact mechanism of FDI quantity and quality on green innovation efficiency

FDI can provide substantial financial support and advanced green technology to aid China’s green innovation activities [37]. Nonetheless, it may yield environmental pressure. Specifically, FDI could affect the absorption level of regional green technology due to the scaling and competition effects and spur local industries to innovate independently in response to ecological consequences, hence influencing GIE.

#### 3.1.1 FDI quantity and green innovation efficiency

The expansion of FDI can bring the same level of technology and capital to each province in China and produce technology spillover to each section through personnel allocation and industrial linkage effect, which is conducive to the development of local green technology, thus promoting the improvement of GIE. It may also squeeze out the market share of domestic enterprises, thus forcing domestic enterprises at the same level to carry out green innovation [46], and promote the improvement of local GIE. Due to variations in technology levels and absorptive capacities across Chinese provinces, the promotion of GIE differs. As Chinese provinces enhance their knowledge accumulation, absorptive capacity, and technology levels, the absorbent effect of Chinese regions on FDI technology spillovers may change accordingly. This could be due to the fact that the absorptive capacity and technological level of China’s provinces must meet a certain threshold before the technology brought in by FDI can be recognized and applied [47]. Based on the absorptive capacity theory [48, 49], the low level of knowledge accumulation (KA) in Chinese provinces poses challenges in absorbing technology brought by FDI expansion in these areas. Consequently, the scaling-up development of FDI creates a substantial crowding-out effect by eliminating many domestic manufacturers, impeding local GIE growth. Therefore, the increase in FDI scale may decelerate the enhancement of the local technology level and absorptive capacity, impeding the advancement of GIE. With the continued enhancement of KA in Chinese provinces, a multitude of absorbable technologies can be acquired through FDI scale expansion. The current stage’s appropriate crowding-out effect of scale expansion will further encourage local green innovation, promoting GIE improvement in each region. Research hypotheses H1a and H2a are proposed based on the preceding analysis.

H1a: FDI quantity can enhance GIE in the provinces of China.
H2a: When the KA of Chinese provinces is low, the amount of FDI can negatively hinder the GIE of each section. However, when KA is high, the FDI_1_ can positively promote the GIE of each section. This suggests a nonlinear threshold effect between the FDI_1_ and the GIE of each province based on its KA.

#### 3.1.2 FDI quality and green innovation efficiency

According to the theory of competition and cooperation [50], FDI_1_ can exert competitive pressure on the host country through the competitive effect. As FDI_2_ increases, the competitive advantage of FDI becomes more substantial. On the one hand, this competitive pressure will stimulate local enterprises to innovate and improve local technological innovation capability through the competitive effect. On the other hand, it will also raise the market entry threshold, drive out inefficient and high-consumption local enterprises, stimulate the enthusiasm of enterprises to carry out green technology activities, and improve the local environmental quality in China [51], thus promoting the improvement of GIE. According to the absorptive capacity theory [48, 49], when KA in Chinese provinces is low, FDI_2_ inhibits the development of local enterprises through the crowding-out effect. As a result, local enterprises are unable to absorb FDI technology well, thus inhibiting the degree of local technology absorption. With the further improvement of KA in China’s provinces, the quality of FDI enables local enterprises to obtain an external economy through the technology spillover effect, which promotes the advancement of local technology absorption level and the improvement of local GIE.

H1b: FDI_2_ can improve the GIE in China.
H2b: When KA of Chinese provinces is low, FDI_2_ will have a negative inhibitory effect on the GIE of each section, while when KA of Chinese provinces is high, FDI_2_ will have a positive promotion effect on the GIE of each section, that is, there is a nonlinear threshold effect between the quality of foreign investment and GIE based on each province’s KA.

### 3.2 An analysis of the mechanism of FDI on green innovation efficiency

FDI can introduce technology and capital into the host nation, promoting its advancements. However, the availability of FDI is restricted, leading to increased competition among local governments seeking to acquire FDI. To secure more FDI, local governments strive to attract investment through costly tax holidays, regulatory exemptions, and substantial incentives [52]; This can even entail the compromise of environmental regulations [34], as per the theory of local government competition. On the contrary, FDI frequently adopts consistent and rigorous environmental standards, which, upon implementation by the host country, may boost the progress of domestic environmental protection technologies, consequently enhancing local environmental governance [53]. Due to subnational governmental competition, FDI can modulate the extent of environmental regulation. From the financial outlook, the GIE is significantly influenced by the degree of environmental regulation. Improvement in environmental regulations would raise the expenses for the pollution control of domestic establishments. Consequently, the cost effect can result in cutting funds for innovation and negatively impacting the GIE. Contrarily, an innovation compensation effect would lead to a relative profit increase in green technology, which would positively impact the GIE. Therefore, environmental regulation levels significantly determine the GIE.

In conclusion, we can derive hypothesis H3, which states that FDI may impact GIE through environmental regulation levels.

**Fig 1:**
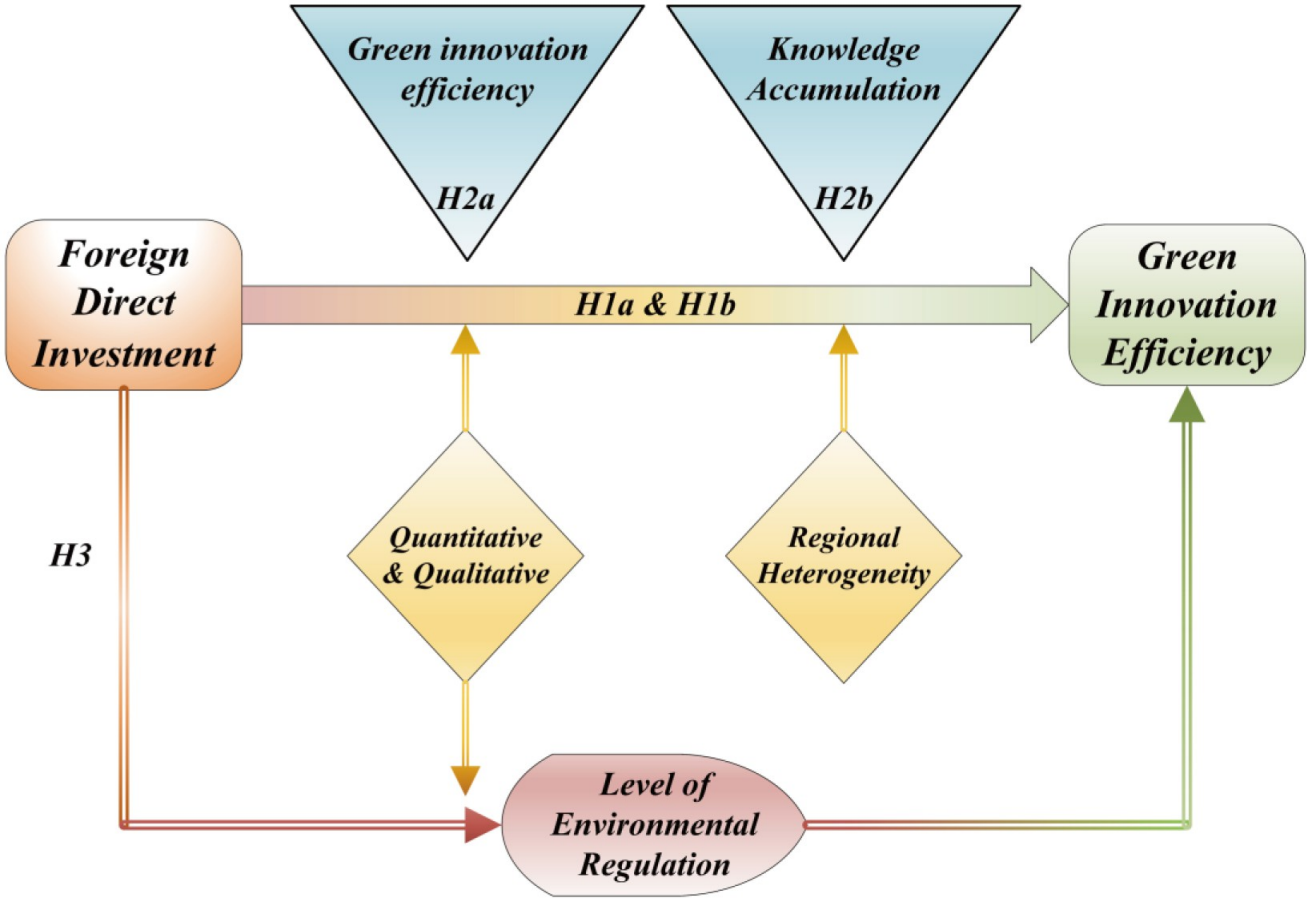
Theoretical framework.

Combined with the above analysis, this paper draws the theoretical framework map, as shown in Fig 1.

## 4. Measurement and description of green innovation efficiency

### 4.1 Super DDF-GML model

Based on the need for research, this paper uses the directional distance function model to measure the GIE of each province in China. The directional distance function model (DDF) is a generalized expression of the radial DEA model, and it is also a method to estimate the relative efficiencies of decision-making units (DMUs) along a predetermined direction vector without radial constraints. However, the standard efficiency model of the directional distance function cannot distinguish the effective DMU. For this problem, Andersen & Petersen (1993) proposed a super-efficiency model, which can further determine the effective DMU [54]. The super-efficiency model of the directional distance function is based on the standard efficiency model by adding the restriction of j≠k [55]. The primary function of the super-efficient directional distance function (SDDF) used in this paper is:

$$\begin{array}{l} \text{max}\;\beta \\ s.t.\sum_{j=1,j\neq k}^{n}\lambda_{j}x_{ij}+\beta g_{xi}\leq x_{ik}\\ \begin{array}{cc} & \sum_{j=1,j\neq k}^{n}\lambda_{j}y_{rj}-\beta g_{yr}\geq y_{rk} \end{array}\\ \begin{array}{cc} & \sum_{j=1,j\neq k}^{n}\lambda_{j}b_{tj}-\beta g_{yt}\leq b_{tk} \end{array}\\ \begin{array}{cc} & \sum_{j=1,j\neq k}^{n}\lambda_{j}=1 \end{array}\\ \begin{array}{cc} & \begin{array}{l} \lambda \geq 0\\ i=1,2\cdots ,m;r=1,2\cdots ,q_{1};t=1,2\cdots ,q_{2};j=1,2\cdots ,n(j\neq k) \end{array} \end{array} \end{array}$$ (1)

Where m is the number of inputs, q1 is the number of expected outputs, and q2 is the quantity of undesired outputs. Considering that the ML index constructed based on the production possibility set of the current period is not transitive, Pastor and Lovell (2005) proposed a GML index containing the production possibility set of all DMUs in each period [56]. GML index has the characteristics of transitivity and circularity, which solves the problem that the traditional ML index has no linear solution and can not compare the efficiency across periods, so this paper chooses the GML index. The GML index takes the global frontier as the reference frontier, where the global frontier refers to the frontier constructed by all phases, and the specific formula is as follows:

$$GML(t-1,t)=\frac{Score_{g}(x_{t},y_{t})}{Score_{g}(x_{t-1},y_{t-1})}$$ (2)

Where *Score*_*g*_(*x*_*t*_, *y*_*t*_) represents the DEA efficiency value derived by (*x*_*t*_, *y*_*t*_) with reference to the global frontier; *Score*_*t*−1_(*x*_*t*−1_, *y*_*t*−1_) represents the DEA efficiency value derived by (*x*_*t*−1_, *y*_*t*−1_) with reference to the global frontier. When the GML of a province is greater than 1, the GIE of the province increases from t to t+1, otherwise it decreases. GIE is the cumulative multiplication of GML index, that is, the GIE of a certain year can be obtained by cumulative multiplication from the base period to the GML of a certain year.

### 4.2 GIE measures and data sources

Based on the existing literature, this paper selects six indicators to construct the input-output index system of GIE, as follows:

1. Capital investment: Concerning the research of Zhuang et al (2022), this paper uses R&D expenditure as a proxy for R&D investment [22].
2. Labor input: Commonly used indicators mainly include R&D personnel and the full-time equivalent of personnel, in which the full-time equivalent of R&D personnel is the number of full-time personnel plus part-time personnel converted according to the workload, which can better reflect the quantity of human resources input in the whole process of regional innovation activities. Therefore, this paper uses the full-time equivalent of R&D personnel to measure the labor input in the process of regional green innovation [37].
3. Energy input: To obtain the total energy consumption of each region that can be measured and compared, this paper converts the consumption of coal, oil, natural gas, electricity, and other energy sources into "ten thousand tons of standard coal" for summation, and selects the total energy consumption after outline to represent the energy input of the region [37].
4. Expected output: measured by two secondary indicators of green patents and sales revenue of new products [57]. Referring to the existing literature [5, 58], this paper uses the quantity of green patent authorization as a substitute variable for green innovation. Sales revenue of new products is usually used to measure the degree of product innovation, so this paper selects the index.
5. Undesired output: carbon emissions. Concerning the research of IPCC (2006), the carbon emission calculation formula used in this paper is shown in Formula 3:

$$c=\sum c_{i}=\sum e_{i}\varepsilon_{i}$$ (3)

Where *c* represents carbon emissions, *c*_*i*_ represents carbon emissions from coal, coke, gasoline, kerosene, diesel, natural gas and cement, *e*_*i*_ is the total consumption of each fossil fuel and cement, and *ε*_*i*_ is the carbon emission factor of each fossil fuel and cement.

The data of the above six indicators are from the CSMAR Database, China Energy Statistics Yearbook, China Statistics Yearbook, and China Research Data Service Platform (CNRDS) from 2011 to 2020, and the specific indicators are described in Table 1.

**Table 1:** Description of relevant indicators of GIE.

	Variables	Obs	Mean	Std. dev.	Min	Max	Unit	Data Source
Labor Input	Full-time equivalent of R&D personnel	300	8.9966	12.3794	0.1157	70.0017	Billion	CSMAR
Capital Input	R&D expenditure	300	3.5310	4.5977	0.0578	25.0000	Ten billion RMB	CSMAR
Energy Input	Total energy consumption	300	1.5156	0.8992	0.1601	4.2456	100 million tons of standard coal	China Energy Statistical Yearbook
Expected Output	Sales revenue of new products	300	5.4900	7.6400	0.0086	44.3000	100 billion RMB	China Statistical Yearbook
	Green patent	300	0.7622	1.0633	0.0031	6.7258	Ten thousand pieces	CNRDS
Unexpected Output	Carbon emissions	300	3.8066	2.6186	0.4242	18.6342	Hundred million tons	China Energy Statistical Yearbook

### 4.3 GIE description

This paper divides the country into two parts from the perspectives of education level and main functional areas. According to the level of education, the government can be divided into two regions: the region with high education levels and the region with low education levels. The specific division method is as follows: by averaging the years of education in the sample period, we can get the average years of teaching in all provinces and cities in China and the national middle years of schooling. The towns and regions longer than the national average are set as areas with high education levels. Otherwise, they are designated as areas with low education levels. According to the National Plan for Main Functional Areas issued by the State Council, the country can be divided into four regions: priority development areas, key development areas, restricted development areas, and prohibited development areas. Because no province has a restricted or prohibited development zone, this paper only divides the whole country into two categories: priority development areas and key development areas.

**Fig 2:**
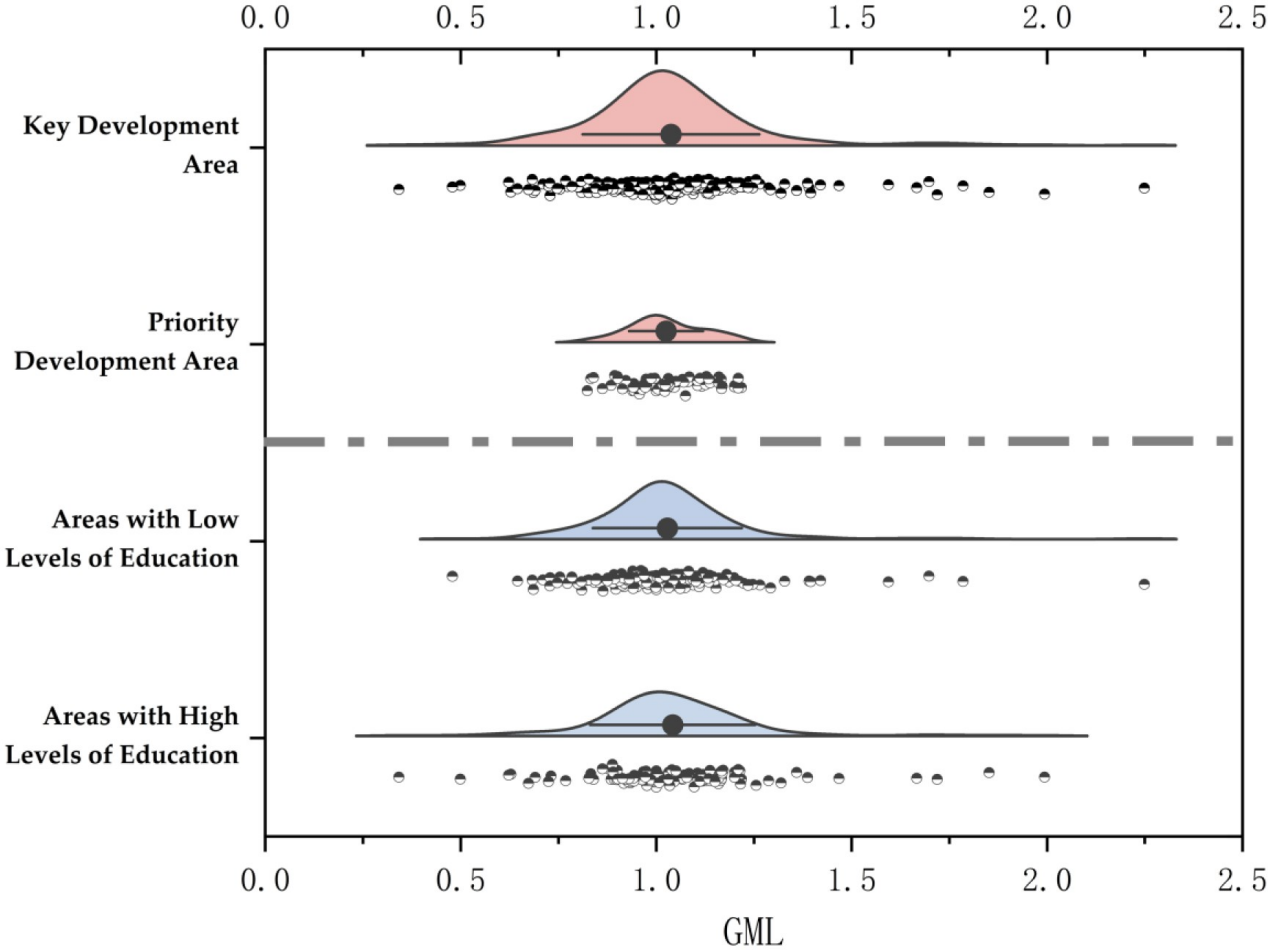
GML distribution of main functional area grouping and education level grouping.

This section describes the GML of China’s provinces and different regional groups from the overall, time, and regional perspectives. It can be seen from Fig 2 that compared with the priority development areas, the distribution of GML in the key development areas is more comprehensive, which indicates that the difference of GML among provinces and cities in the key development areas is more significant. The median of GML in these two regions is greater than 1, which indicates that the GIE in most regional provinces is improving. The distribution of GML in low and high-education areas is roughly equivalent, and the GML in both regions is greater than 1, indicating that most of the GIE is increasing in both regions.

**Fig 3:**
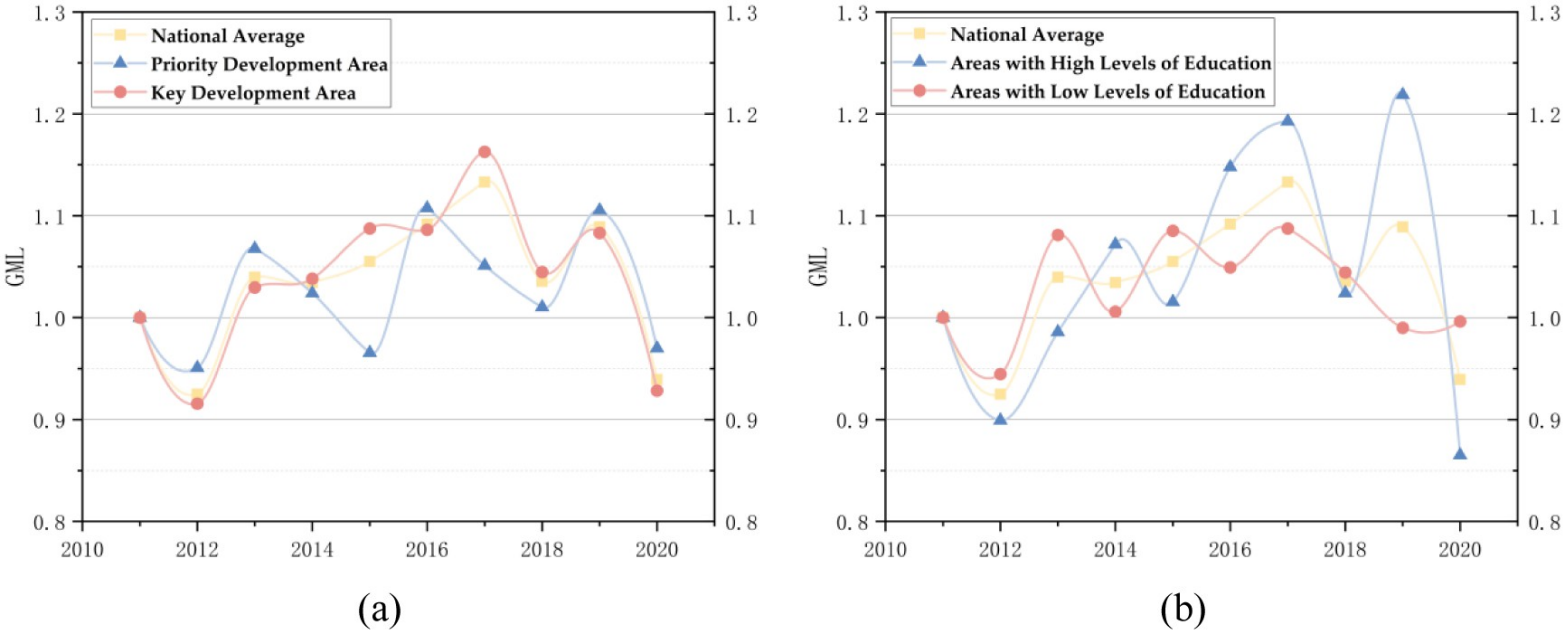
GML Change of main functional area (a) and education level group (b) from 2011 to 2020.

Fig 3 can be obtained by averaging the GML of different regions and the whole country from 2011 to 2020. It can be seen from Fig 3 that the national average GML was less than 1 in 2012 and 2020, which indicates that the national GIE has declined to a certain extent in these two years. The change in the national average GML can be divided into two stages: the rising fluctuation stage from 2012 to 2017 and the declining phase from 2017 to 2020. In the increasing degree of change, the national GIE is constantly improving, and the growth rate is faster and faster, which may be because the state pays more attention to green innovation and increases the support for sustainable innovation; in the declining stage of fluctuation, the growth rate of national GIE is slowing down, which may be because the development of national green innovation has reached a particular bottleneck. By 2020, the GML will be less than 1, possibly due to the poor flow of innovation resources caused by the epidemic and the significant decline in innovation output. The GML of the priority development area has fluctuated, and the upward trend is not apparent, indicating that the GIE in this area has changed significantly. The change of GML in key development areas is similar to the national average GML, which can be divided into two stages: the rising stage of fluctuation from 2012 to 2017 and the declining stage of change from 2017 to 2020. In the previous step, the GIE in the region constantly improves, and the growth rate is accelerating. In the latter setting, the growth rate of GIE in the part is slowing down or even declining. The change of GML in high and low education areas can be divided into two stages: the rising stage of fluctuation from 2012 to 2017 and the fluctuation stage from 2017 to 2020.

**Fig 4:**
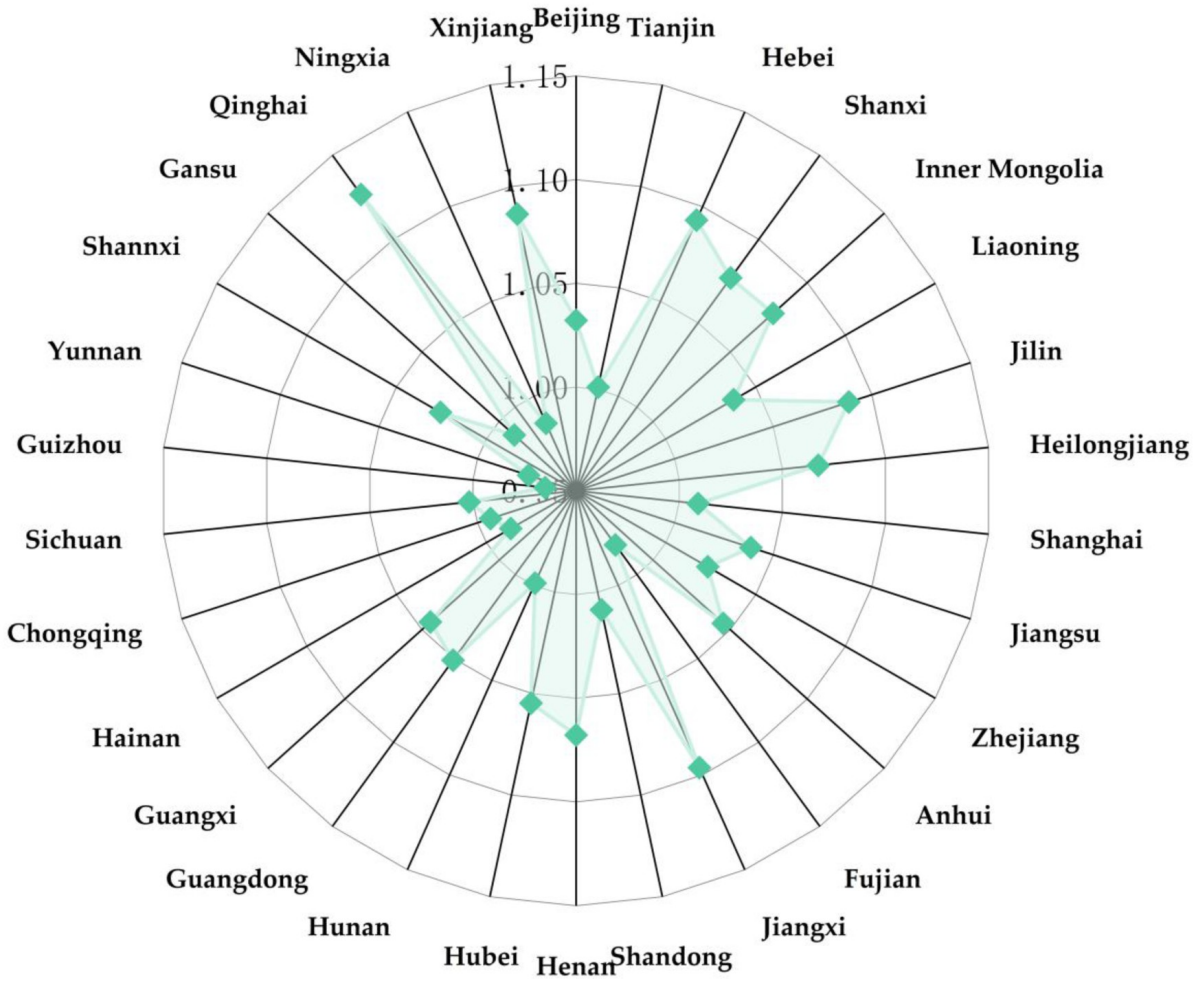
GML distribution in all provinces of China.

Fig 4 can be obtained by averaging the GML of all provinces in China from 2011 to 2020. It can be seen from Fig 4 that the GML of seven regions and cities is less than 1, namely Gansu, Ningxia, Chongqing, Yunnan, Guizhou, Hunan, and Fujian. The GIE of these provinces is declining, which may be due to the need for more innovation talents, imperfect incentive mechanisms, and insufficient motivation for technological research and development in these provinces and cities. The GIE in most other towns and regions is constantly improving, with the fastest improvement in Qinghai, Jiangxi, and other areas.

## 5. Model settings and variable descriptions

### 5.1 Model settings

#### 5.1.1 Benchmark regression model

To explore the impact of the quantity and quality of FDI on the GIE of Chinese provinces, this paper selects the mixed OLS regression, individual fixed effects model, and two-way fixed effects model to identify the impact. Among them, the two-way fixed effect model constructed in this paper is as follows:

$$GIE_{it}=\eta_{1}FDI_{it}+\eta_{2}\sum X_{it}^{\prime }+\mu_{i}+\gamma_{t}+\varepsilon_{it}$$ (4)

Where *FDI* is FDI_1_ and FDI_2_; *X* represents other control variables, including economic development level (lnED), foreign trade level (lnFT), R & D level (lnRD), marketization level (lnMA), urban-rural income gap (lnURI) and financial development (lnFD). *η*_0_ is the constant term, *η* is the regression coefficient of FDI, *η*_2_ is the regression coefficient of control variables, *μ* represents the individual fixed effect, *γ* represents the time fixed effect, and *ε* is the random error term.

#### 5.1.2 Mediating effect model

To further explore the relationship between the quantity and quality of FDI and GIE, this paper uses the mediating effect model to explore the impact mechanism of FDI on GIE. According to the previous mechanism analysis, FDI can affect the GIE through environmental regulation, in which the intermediary variable is the level of environmental regulation (ER). Therefore, this paper constructs the following model using the step-by-step method [59]:

$$\begin{array}{l} GIE_{it}=cFDI_{it}+\eta_{2}\sum X_{it}^{\prime }+\mu_{i}+\gamma_{t}+\varepsilon_{it}\\ ER_{it}=aFDI_{it}+\eta_{2}\sum X_{it}^{\prime }+\mu_{i}+\gamma_{t}+\varepsilon_{it}\\ GIE_{it}=c^{*}FDI_{it}+bER_{it}+\eta_{2}\sum X_{it}^{\prime }+\mu_{i}+\gamma_{t}+\varepsilon_{it} \end{array}$$ (5)

The above equations examine whether the mediating variables are critical paths of influence. Among them, the first formula measures the impact of FDI on GIE, that is, whether the core explanatory variables affect the explained variables; the second formula measures the effects of FDI on the ER, that is, whether the explanatory variables affect the intermediary variables; In the third formula, both the explanatory variables and the mediating variables are included in the equation, and whether the mediating variables have an impact on the explained variables is discussed. By comparing the coefficients c and c*, we can judge whether the mediation effect is significant. The size and proportion of the mediation effect to the total impact and the balance of the mediation effect to the actual result can also be considered according to Formula 5.

#### 5.1.3 Threshold model

The impact of FDI on GIE in China’s provinces may change with the change of GIE; that is, the effect of FDI on GIE may be nonlinear. This impact may also be affected by KA in different provinces of China; that is, there are changes in the effects of FDI on GIE at varying KA. To effectively test this nonlinear effect and avoid the error caused by manual grouping, this paper uses the Hansen nonlinear single threshold model [60] to explore the nonlinear impact of FDI on GIE of Chinese provinces. Refer to existing literature [61], a piecewise function with KA and GIE as threshold variables is constructed as follows.


$$GIE_{it}=\eta +\eta_{11}FDI_{it}I(q_{it}<\gamma )+\eta_{12}FDI_{it}I(q_{it}\geq \gamma )+\eta_{2}\sum X_{it}^{\prime }+u_{i}+\varepsilon_{it}$$ (6)

*q*_*it*_ is the threshold variable, specifically the KA and GIE. *γ* is the threshold to be estimated. The individual effect is represented by the parameter *u*_*i*_.

### 5.2 Variable index design

#### 5.2.1 Explanatory variables

FDI_1_ and FDI_2_. In this paper, the actual use of foreign capital is used as a proxy variable for the FDI_1_, and the US dollar-denominated FDI is converted into RMB according to the annual exchange rate of RMB to US dollar [62, 63]. Referring to relevant literature [64], this paper uses the following formula to calculate the FDI_2_:

$$FDI_{2}=\frac{FDI_{it}/FDI_{t}}{GDP_{it}/GDP_{t}}$$ (7)

Where *FDI*_*it*_ represents the FDI_1_ of the ith province in period t, *FDI*_*t*_ represents the FDI_1_ of 30 provinces and cities in China in period t, *GDP*_*it*_ represents the GDP of the ith province in period t and *GDP*_*t*_ represents the GDP of 30 provinces and cities in China.

#### 5.2.2 Explained variable

Green Innovation Efficiency (GIE). The specific calculation can be found in Chapter 4.

#### 5.2.3 Mediating variable

Environmental Regulation Level (ER). This paper uses two methods to measure the ER, ER_1_ and ER_2_, respectively. (1) Referring to the relevant literature [25, 65], and considering that the environmental effect can not be composed of a single pollutant but needs a more comprehensive index, this paper uses the entropy method to calculate the ER_1_ based on the data of three wastes emissions in each province. The three wastes refer to the discharge of major pollutants such as solid waste, wastewater, and waste gas. The specific calculation steps are as follows: standardize the three pollutants, calculate the weight of each pollutant, and obtain the environmental regulation level through the product of weight and standardization. The consequences of waste solid, wastewater and waste gas are 0.34, 0.30, and 0.36, respectively. (2) This paper uses the ratio of emission tax to industrial-added value to measure ER_2_.

#### 5.2.4 Threshold variables

KA and GIE. To effectively analyze whether FDI_1_ and FDI_2_ have nonlinear effects on China’s GIE under different KA and GIE in other provinces of China, this paper constructs two indicators of KA and GIE, the latter of which has been obtained in the previous form. Referring to the previous literature [66], using patents to measure knowledge production is a standard method in academia. Hence, this paper selects the number of patent applications to measure the level of new knowledge production. Because of the need to measure KA, this paper adopts the perpetual inventory method proposed by Goldsmith to calculate, in which the number of patent applications is used as a proxy indicator of new knowledge production. The specific calculation formula is as follows:

$$A_{it}=(1-d)A_{i,t-1}+P_{i,t-1}$$ (8)

Where *A*_*it*_ denotes the stock of knowledge in the ith province at the beginning of period t, *A*_*i*,*t*−1_ denotes the stock of knowledge in the ith province at the beginning of period t-1, *P*_*i*,*t*−1_ denotes the newly produced knowledge in the ith province at the beginning of period t-1, and d is the depreciation rate. Referring to the existing literature [67], the calculation formula of knowledge stock in the base period of this paper is as follows:

$$A_{i0}=\frac{P_{i1}}{g_{i}+d}$$ (9)

Where *g*_*i*_ is the average annual growth rate of the ith province, referring to the existing literature [37], a depreciation rate of 15% is used for the measurement in this paper.

#### 5.2.5 Control variables

The economy, science, technology, market, and finance influence China’s provincial GIE. Therefore, this paper chooses the following six control variables according to the previous relevant literature and data availability, namely, the level of economic development (lnED), the level of foreign trade (lnFT), the level of research and development (lnRD), the degree of marketization (lnMA), urban-rural income gap (lnURI) and the level of financial development (lnFD). The level of economic development is expressed by GDP per capita [21], and the level of foreign trade is measured by the ratio of total imports and exports to GDP [22]. The level of R&D is measured by the ratio of internal expenditure of R&D funds to GDP, and the level of marketization is measured by the general index of China’s marketization [68]. The level of urban-rural income gap is measured by the Theil index [40], and the level of financial development is measured by the ratio of loan balance to deposit balance of banking financial institutions in each province.

### 5.3 Data source and description

The data for all indicators used in this paper come from the 2011–2020 China Statistical Yearbook, China Energy Statistical Yearbook, CSMAR database, CNRDS, China Marketization Index Report, and provincial statistical yearbooks. See Table 2 for a description of the specific data.

**Table 2:** Description of relevant variables.

	Variables	Abbreviation	Obs	Mean	Std.dev.	Min	Max	Unit
explanatory variables	Quantity of FDI	FDI_1_	300	5.3825	5.2009	0.0030	22.573	Billion RMB.
	Quality of FDI	FDI_2_	300	0.9085	0.7669	0.0058	5.2580	\
Explained variable	Green innovation efficiency	GIE	300	1.1187	0.3768	0.2147	2.3865	\
Mediating variable	Level of environmental regulation	ER	300	0.7676	0.1961	0.1052	1.0663	\
Threshold variable	Level of knowledge accumulation	KA	300	39.168	58.205	0.1836	342.78	Ten thousand pieces
Control variables	Level of economic development	ED	300	5.6386	2.7306	1.6413	16.489	Ten thousand RMB
	Level of foreign trade	FT	300	0.2526	0.2672	0.0072	1.4577	\
	R&D level	RD	300	0.0167	0.0113	0.0041	0.0644	\
	Marketization level	MA	300	7.9253	1.8901	3.3600	12.107	\
	Level of urban-rural income gap	URI	300	0.0887	0.0389	0.0180	0.2020	\
	Level of financial development	FD	300	0.7891	0.1436	0.4483	1.1641	\

## 6. Empirical analysis

### 6.1 Benchmark regression analysis

Table 3 reports the regression results based on mixed OLS regression, individual fixed effects model, and two-way fixed effects model. Columns (1), (3), and (5) report the impact of FDI_1_ on GIE in Chinese provinces, and columns (2), (4), and (6) report the effects of FDI_2_ on GIE in Chinese areas.

**Table 3:** Benchmark regression results.

Variables	Pooling OLS	Pooling OLS	Region-fixed effects	Region-fixed effects	Two-way fixed effects	Two-way fixed effects
	(1)	(2)	(3)	(4)	(5)	(6)
FDI_1_	0.0272***		0.0334***		0.0235***	
	(4.32)		(5.95)		(3.37)	
FDI_2_		0.0126		0.0790**		0.1355***
		(0.35)		(2.37)		(4.09)
LnED	-0.0963	0.0151	-0.4374***	-0.2712***	-0.7381***	-0.7543***
	(-0.95)	(0.15)	(-4.38)	(-2.69)	(-4.64)	(-4.85)
LnFT	-0.1464***	-0.1175***	-0.0543*	-0.0177	0.0752	0.0978
	(-4.37)	(-3.47)	(-1.68)	(-0.52)	(0.92)	(1.21)
LnRD	-0.1899***	-0.1540**	-0.1605**	-0.1180*	-0.4135***	-0.4352***
	(-2.65)	(-2.09)	(-2.48)	(-1.74)	(-2.90)	(-3.08)
LnMA	0.3029*	0.5306***	0.0193	0.2588*	0.2736	0.2191
	(1.93)	(3.43)	(0.13)	(1.75)	(1.37)	(1.10)
LnURI	-0.2197***	-0.1150	-0.2742***	-0.0837	-0.2981	-0.2616
	(-2.66)	(-1.29)	(-3.73)	(-1.04)	(-0.78)	(-0.70)
LnFD	-0.1415	-0.2779*	-0.4033***	-0.5593***	-0.0671	-0.0899
	(-0.99)	(-1.93)	(-3.07)	(-4.13)	(-0.27)	(-0.37)
_cons	-0.2743	-1.3730	4.0573***	2.5688*	5.6920***	5.9686***
	(-0.22)	(-1.07)	(3.18)	(1.96)	(2.94)	(3.11)
City	NO	NO	YES	YES	YES	YES
Year	NO	NO	NO	NO	YES	YES
R^2^	0.1222	0.0665	0.2094	0.1277	0.4128	0.4244
N	300	300	300	300	300	300
F test			10.20***	8.70***	11.44***	13.97***
Hausman test					57.85***	43.35***

Note: t statistics in parentheses

* p < 0.1,

** p < 0.05,

*** p < 0.01

The fixed effects model is optimal, as can be seen from the fact that the F-test is significantly positive and the Hausman test is passed substantially. From the regression results in column (5), it can be seen that the coefficient of FDI_1_ is 0.0235. It is significant at the 1% level, which indicates that every 1% increase in FDI leads to a rise in GIE of China’s provinces and cities by 0.0235%. The goodness of fit of this column is 0.4128, which indicates that the FDI_1_ can explain the changes in GIE of China’s provinces to a greater extent. The regression results from column (6) show that the coefficient of FDI_1_ is 0.1355 and is significant at the 1% level, which indicates that every 1% increase in the FDI_2_ leads to a rise in gie of China’s provinces and cities by 0.1355%. The goodness of fit of this column is 0.4244, which indicates that the FDI_2_ can explain the changes in GIE of China’s provinces to a greater extent. According to the above analysis, FDI_1_ and FDI_2_ positively impact GIE. In this regard, the above study proves hypotheses H1a and H1b.

### 6.2 Endogenous test

The issue of endogeneity, stemming from the omission of variables and reverse causation, has been tackled in this study by implementing a two-way fixed effects model. Nevertheless, there remains a possibility of endogeneity issues. To address potential endogeneity problems, this research employs generalized moment estimation SYS-GMM and two-stage least squares (2SLS) for regression analysis. Table 4 presents the regression results. The SYS-GMM approach alleviates the issues of individual heterogeneity, measurement error, and omitted variable bias using lagged instrumental variables and difference-in-differences operations. In this study, we have selected the lagged instrumental variable for the regression of the method as the lag 1 period of the GIE, designated as L.GIE. The 2SLS technique necessitates identifying a suitable instrumental variable that is correlated and exogenous to tackle endogeneity issues. In this paper, we have selected the interaction term and time variable of the number of Christian church students below secondary school and the number of church primary school students per 10,000 people with OFDI in 1919 as instrumental variables, which we have named *iv1*, *iv2*, and *iv3*. We have chosen these three instrumental variables based on the correlation between them. The number of Christian students in 1919 can reflect the level of externality acceptance within a region and, therefore, is correlated with FDI. There is typically a correlation between the time variable and FDI. The number of Christian students in 1919 and the time variable show no correlation with the disturbance term from 2011–2020.

**Table 4:** Endogenous regression results.

Model	SYS-GMM		2SLS			
variables	(1)	(2)	(3)	(4)	(5)	(6)
	GIE	GIE	FDI_1_	GIE	FDI_2_	GIE
L.GIE	0.6823***	0.7861***				
	(4.44)	(7.13)				
FDI_1_	0.0171**			0.0849***		
	(2.01)			(5.38)		
FDI_2_		0.0845*				-0.5181***
		(1.75)				(-3.18)
*iv1*			0.9013***			
			(7.20)			
*iv2*					0.0242**	
					(2.60)	
*iv3*					-0.662***	
					(-4.28)	
LnED	0.1788	0.4758**	4.3300***	-0.6874***	0.0156	-0.1688
	(1.23)	(2.19)	(4.62)	(-5.25)	(0.09)	(-1.17)
LnFT	-0.2244**	-0.2099**	0.1087	-0.0925**	-0.1474**	-0.1444***
	(-2.36)	(-2.33)	(0.34)	(-2.48)	(-2.56)	(-3.21)
LnRD	-0.1056	-0.0358	0.9163	-0.2171***	-0.0129	-0.1664*
	(-0.50)	(-0.12)	(1.46)	(-2.95)	(-0.11)	(-1.73)
LnMA	0.3224	0.0930	4.5139***	-0.4738**	0.7166***	0.9398***
	(1.20)	(0.29)	(3.00)	(-2.22)	(2.66)	(4.01)
LnURI	-0.0616	0.1318	0.5291	-0.4558***	-0.7910***	-0.6154***
	(-0.45)	(1.14)	(0.66)	(-4.74)	(-5.73)	(-3.29)
LnFD	-0.0392	-0.4628	-4.2205***	-0.1693	0.2227	-0.3745**
	(-0.12)	(-1.61)	(-3.36)	(-1.06)	(0.92)	(-1.97)
_cons	-3.3492*	-5.4804*	-47.041***	6.4967***	130.266***	-0.5181***
	(-1.74)	(-1.77)	(-3.96)	(4.12)	(4.30)	(-3.18)
N	270	270	300	300	300	300
AR(1)	0.056	0.043				
AR(2)	0.673	0.595				
Hansen test	20.77	22.75				
Anderson canon. corr. LM statistic				46.479***		24.850***
Cragg-Donald Wald F statistic				51.883		13.141
Sargan statistic						26.551***

Note: t statistics in parentheses

* p < 0.1,

** p < 0.05,

*** p < 0.01

Table 4’s columns (1) and (2) indicate that AR(1) is significant, AR(2) is not substantial, and the Hansen test is not important, which implies that the initial hypothesis, which stated that all instrumental variables are exogenous, cannot be rejected. The coefficients for *iv1*, *iv2*, and *iv3* are all statistically significant, as displayed in columns (3)-(6) of Table 4. This suggests a strong correlation between these instrumental and endogenous independent variables. Furthermore, the F-statistic test value is greater than 10, indicating a lack of weak instrumental variables among all selected instrumental variables. Moreover, it has been observed that all p-values of the LM statistic statistics in the Anderson canonical correlation are below 0.1, thereby rejecting the initial non-identifiability hypothesis. Consequently, instrumental variables *iv1*, *iv2*, and *iv3* are valid.

### 6.3 Robustness test

This paper uses two methods to avoid possible errors in using the SDDF-GML model: replacing the explanatory variables and shrink-tail processing. Specifically, replacing the explanatory variables means that this paper recalculates the GIE using the MinDW-GML model and performs the regression; the reduced-tail treatment means that the values of each variable that are smaller than the first percentile are replaced with the first percentile, and the importance of each variable that is larger than the ninety-ninth percentile are replaced with the ninety-ninth percentile and then performed the regression, respectively. According to the results in Table 5, this paper finds that FDI_1_ and FDI_2_ can still significantly impact GIE. Hence, the results of the benchmark regression are robust.

**Table 5:** Regression results after replacement of explanatory variables and shrinkage treatment.

Variables	Replace the Explained Variable		Shrinkage Treatment	
	(1)	(2)	(3)	(4)
FDI_1_	0.0036***		0.0248***	
	(2.80)		(3.50)	
FDI_2_		0.0211***		0.1441***
		(3.44)		(4.07)
LnED	-0.0873***	-0.0904***	-0.7600***	-0.7692***
	(-2.97)	(-3.14)	(-4.65)	(-4.82)
LnFT	0.0070	0.0105	0.0740	0.0915
	(0.46)	(0.70)	(0.86)	(1.07)
LnRD	-0.0286	-0.0321	-0.3936***	-0.4080***
	(-1.09)	(-1.23)	(-2.78)	(-2.90)
LnMA	0.0217	0.0131	0.2707	0.2149
	(0.59)	(0.36)	(1.36)	(1.08)
LnURI	-0.0005	0.0045	-0.4091	-0.4293
	(-0.01)	(0.06)	(-1.10)	(-1.16)
LnFD	-0.0005	-0.0040	-0.0481	-0.0952
	(-0.01)	(-0.09)	(-0.20)	(-0.39)
_cons	1.7433***	1.7890***	5.7622***	5.8467***
	(4.87)	(5.03)	(2.94)	(3.02)
City	YES	YES	YES	YES
Year	YES	YES	YES	YES
R^2^	0.3228	0.3330	0.4109	0.4202
N	300	300	300	300
F test	9.79***	12.37***	11.25***	13.75***
Hausman test	37.23***	23.13***	60.56***	46.33***

Note: t statistics in parentheses

* p < 0.1,

** p < 0.05,

*** p < 0.01

### 6.4 Further discussion

#### 6.4.1 Analysis of mediating effect

Referring to Jiang, (2022), this paper adopts theoretical proof and empirical tests to prove the mediating effect of ER [69], demonstrated in the previous section from an academic perspective. In this section, the paper empirically presents the mediating effect of ER using the mediating effect model. Table 6 reports the empirical results with ER as the mediating variable. Among them, column (1) and (3) verifies the existence of the transmission path of "FDI_1_-ER"; columns (2) and (4) jointly confirms the presence of the transmission path of "FDI_2_-ER".

**Table 6:** Regression results of mediated effects model.

Variables	(1)	(2)	(3)	(4)
	ER_1_	ER_1_	ER_2_	ER_2_
FDI_1_	0.0039***		0.3147***	
	(2.62)		(3.34)	
FDI_2_		0.0183**		1.2026***
		(2.57)		(2.64)
LnED	-0.2025***	-0.1986***	0.3281	1.0948
	(-5.98)	(-5.94)	(0.15)	(0.51)
LnFT	0.0287	0.0320*	-0.8886	-0.6540
	(1.65)	(1.84)	(-0.80)	(-0.59)
LnRD	0.0266	0.0257	-5.2031***	-5.0956***
	(0.88)	(0.85)	(-2.70)	(-2.62)
LnMA	-0.0218	-0.0284	-7.7666***	-8.1355***
	(-0.51)	(-0.66)	(-2.87)	(-2.97)
LnURI	0.0029	0.0159	9.2344*	10.7694**
	(0.04)	(0.20)	(1.79)	(2.09)
LnFD	-0.0917*	-0.0950*	-4.1082	-4.3398
	(-1.77)	(-1.83)	(-1.25)	(-1.30)
_cons	3.1365***	3.1461***	18.8622	16.6287
	(7.38)	(7.38)	(0.70)	(0.61)
City	YES	YES	YES	YES
Year	YES	YES	YES	YES
*N*	300	300	300	300
*R* ^2^	0.9435	0.9434	0.7938	0.7906
F test	105.22***	9.15***	20.17***	19.35***

Note: t statistics in parentheses

* p < 0.1,

** p < 0.05,

*** p < 0.01

The regression results in columns (1) and (3) show that the coefficient of FDI_1_ on ER_1_ and ER_2_ is significantly positive, indicating that the FDI_1_ substantially contributes to the level of environmental regulation. The regression results in columns (2) and (4) show that the coefficient of FDI_2_ on ER_1_ and ER_2_ is significantly positive, indicating that the quality of FDI substantially contributes to the level of environmental regulation. This may be because foreign firms introduced by FDI usually come from countries or regions with more stringent environmental regulations. To comply with local environmental requirements and obtain government support, they are more inclined to promote green innovation and adopt environmentally friendly technologies and equipment in their production processes. This push effect of environmental regulations motivates firms to improve and increase the GIE continuously. In this regard, the above analysis proves hypothesis H3.

#### 6.4.2 Analysis of regional heterogeneity

There is a positive impact between FDI on GIE, will this impact be affected by regional differences? In this section, China is divided into two categories: high education level regions and low education level regions, and priority development zones and main functional zones, to empirically analyze the impact of regional differences and explore the reasons for them. Table 7 reports the regression results of regional heterogeneity. Columns (1) (2) (3) and (4) report the regression results for the subgroup of education level, and columns (5) (6) (7) and (8) report the regression results for the main functional areas. From the regression results in columns (1) and (3), the coefficients on the FDI_1_ are all positive and all significant at the 5% level. From the regression coefficients, the positive effect of the number of FDI on GIE is significantly higher in regions with low education level than in regions with high education level. And from the regression results in columns (3) and (4), the quality of FDI in low education level regions has a significantly higher impact on GIE than in high education level regions.

**Table 7:** Regression results of regional heterogeneity analysis.

Variables	Subgroups by Level of Education				Main Functional Area Grouping			
	Areas with High Levels of Education		Areas with Low Levels of Education		Priority Development Areas		Key Development Area	
	(1)	(2)	(3)	(4)	(5)	(6)	(7)	(8)
FDI_1_	0.0174**		0.0361**		-0.0162***		0.0465***	
	(2.10)		(2.53)		(-3.35)		(3.42)	
FDI_2_		0.0997***		0.3388***		-0.0558*		0.1885***
		(2.85)		(3.67)		(-1.99)		(2.64)
LnED	-0.5753**	-0.5935***	-0.6322**	-0.5322*	0.2631	0.1547	-1.0740***	-0.9798***
	(-2.46)	(-2.68)	(-2.31)	(-1.97)	(0.91)	(0.49)	(-5.62)	(-5.15)
LnFT	-0.0477	-0.0087	0.0328	0.0286	-0.3985*	-0.4431*	0.0957	0.1282
	(-0.34)	(-0.06)	(0.28)	(0.25)	(-1.82)	(-1.91)	(1.00)	(1.34)
LnRD	-0.4985*	-0.5531**	-0.2529	-0.3625*	0.6512***	0.6855***	-0.6477***	-0.6058***
	(-1.94)	(-2.17)	(-1.29)	(-1.86)	(3.48)	(3.39)	(-3.92)	(-3.64)
LnMA	0.5687	0.4623	0.2425	0.2022	0.8020**	0.7155**	0.2114	0.1910
	(1.66)	(1.36)	(0.71)	(0.61)	(2.38)	(2.02)	(0.89)	(0.79)
LnURI	-1.0945**	-1.0222**	0.5134	0.2200	0.2856	0.0964	-0.0917	-0.01473
	(-2.39)	(-2.32)	(0.62)	(0.27)	(0.80)	(0.26)	(-0.15)	(-0.02)
LnFD	-0.0732	-0.0950	-0.0256	-0.0950	0.3530	0.2822	-0.5001	-0.5542
	(-0.24)	(-0.32)	(-0.06)	(-0.23)	(1.15)	(0.84)	(-1.31)	(-1.43)
_cons	0.8648	1.2608	6.9221*	4.7134	-0.3057	0.5358	8.3800***	7.8440***
	(0.32)	(0.48)	(1.97)	(1.33)	(-0.10)	(0.16)	(3.50)	(3.17)
City	YES	YES	YES	YES	YES	YES	YES	YES
Year	YES	YES	YES	YES	YES	YES	YES	YES
R^2^	0.6190	0.6320	0.3100	0.3426	0.7018	0.6656	0.4926	0.4799
N	130	130	170	170	80	80	220	220
F test	13.72***	17.02***	2.37***	2.38***	12.45***	10.11***	12.44***	14.17***

Note: t statistics in parentheses

* p < 0.1,

** p < 0.05,

*** p < 0.01

There are three possible reasons for this: (1) Complementary technology and knowledge. Regions with a low level of education have a relative lack of advanced technology and knowledge, and FDI can fill this knowledge and technology gap by way of technology transfer, providing more opportunities and capabilities for green innovation. In regions with a low level of education, the introduction of technology by FDI can enhance the GIE to a greater extent. (2) Introduction of funds and resources. Regions with a low level of education usually face a shortage of funds and resources, which limits the development of green innovation. Funds and resources brought in by FDI can fill this gap and provide local enterprises with more innovative support and conditions. The impact of FDI on the GIE is more significant in the case of shortage of funds and resources. (3) Market and competitive pressure: Regions with low education levels tend to face relatively low market and competitive pressure. The introduction of FDI can bring more market competition and pressure to stimulate the innovation drive and willingness of local enterprises. In regions with low education levels, the impact of FDI on GIE is greater because it can push enterprises to pay more attention to environmental protection and sustainable development and improve GIE. To summarize, the impact of FDI on GIE is more significant in regions with high education level due to the improvement of talent quality and innovation consciousness. In regions with low education levels, the impact of FDI on GIE is more pronounced relative to the improvement of existing resources and market environment.

From the regression results in columns (5), (6), (7) and (8), the regression coefficients of FDI quantity and quality are significantly negative in the Priority Development Zones, while the regression coefficients of FDI quantity and quality are significantly positive in the Focused Development Zones. This indicates that the quantity and quality of FDI hinders the improvement of GIE in the priority development zones, while in the key development zones, the quantity and quality of FDI promotes the improvement of GIE.

#### 6.4.3 Threshold effect analysis

*6*.*4*.*3*.*1 Threshold effect significance test*. Based on the threshold variables of GIE and ER, this paper conducts the significance test of the threshold effect of the quantity and quality of FDI, respectively, and the test results are shown in Tables 8 and 9. According to the test results, the single-threshold threshold effect of FDI_1_ and FDI_2_ is significant at the 1% level for different threshold variables. In this regard, according to the Hausman threshold theory, it can be preliminarily concluded that there is a non-linear effect of the quantity and quality of FDI on GIE.

**Table 8:** Significance test of single threshold effect of quantity and quality of FDI.

TV	EV	RSS	MSE	Fstat	Prob	Crit10	Crit5	Crit1
GIE	FDI_1_	9.5027	0.0328	82.43	0.0000	15.7385	20.5311	32.0948
GIE	FDI_2_	8.0913	0.0279	138.72	0.0000	14.4888	17.7506	26.1786
KA	FDI_1_	10.2373	0.0353	55.70	0.0000	19.4235	22.4780	29.3537
KA	FDI_2_	10.3322	0.0356	45.74	0.0010	20.9381	23.9107	30.8877

**Note**: TV is the threshold variable and EV is the explanatory variable

**Table 9:** Single thresholds and confidence intervals for quantity and quality of FDI.

TV	EV	Estimated threshold	95% Confidence Interval	
GIE	FDI_1_	1.5664	1.5481	1.5693
GIE	FDI_2_	1.5404	1.5222	1.5481
KA	FDI_1_	9.1478	8.8997	9.3168
KA	FDI_2_	9.1478	8.8997	9.3168

*6*.*4*.*3*.*2 Analysis of regression results for threshold effects*. To further explore the specific characteristics of the nonlinear relationship, this paper makes a threshold effect regression on the quantity and quality of FDI, and the results are shown in Table 10. The threshold variable in columns (1) and (2) is the GIE, and the threshold variable in columns (3) and (4) is the level of knowledge accumulation. The core explanatory variable in columns (1) and (3) is FDI_1_, and the core explanatory variable in columns (2) and (4) is FDI_2_.

**Table 10:** Parameter estimation results of FDI quantity and quality model.

TV	GIE	GIE	KA	KA
EV	FDI_1_	FDI_2_	FDI_1_	FDI_2_
Variables	(1)	(2)	(3)	(4)
GIE<1.5664	0.0192***			
	(3.10)			
GIE≥1.5664	0.0722***			
	(8.57)			
GIE<1.5404		0.0797***		
		(2.87)		
GIE≥1.5404		0.5085***		
		(11.68)		
KA<9.1478			-0.1331***	-0.3341***
			(-5.70)	(-4.15)
KA≥9.1478			0.0196***	0.1352***
			(3.05)	(4.38)
LnED	-0.7436***	-0.6324***	-0.6341***	-0.7167***
	(-5.28)	(-4.92)	(-4.32)	(-4.95)
LnFT	0.0469	0.0652	0.0631	0.0797
	(0.65)	(0.98)	(0.84)	(1.05)
LnRD	-0.5592***	-0.5706***	-0.6831***	-0.6181***
	(-4.40)	(-4.87)	(-5.01)	(-4.59)
LnMA	0.3142*	0.2210	0.1681	0.1280
	(1.77)	(1.35)	(0.91)	(0.69)
LnURI	-0.6961**	-0.6066*	-0.3104	-0.2938
	(-2.04)	(-1.96)	(-0.88)	(-0.84)
LnFD	-0.2928	-0.4434**	-0.2731	-0.2910
	(-1.35)	(-2.20)	(-1.21)	(-0.84)
_cons	3.9898**	3.1541*	3.5822**	4.8654***
	(2.31)	(1.97)	(1.99)	(2.71)
R^2^	0.5427	0.6107	0.5074	0.5028
N	300	300	300	300
F test	14.77***	13.53***	17.34***	13.53***

Note: t statistics in parentheses

* p < 0.1,

** p < 0.05,

*** p < 0.01

The regression results of column (1) show that when the GIE of China’s provinces and cities is less than 1.5664, the regression coefficient of FDI_1_ to GIE is 0.0192, which is significant at the 1% level, indicating that the expansion of FDI can significantly improve the GIE of China’s provinces and cities. With the continuous improvement of GIE in China’s towns and regions, the regression coefficient of FDI_1_ on GIE is 0.0722 after exceeding the threshold value. It is significant at 1%, indicating that the expansion of FDI substantially impacts GIE. The regression results of column. (2) show that when the GIE of China’s provinces and cities is less than 1.5404, the regression coefficient of FDI_2_ on GIE is 0.0797, which is significant at the level of 1%, indicating that the improvement of the quality of FDI can significantly promote the advancement of GIE. With the rise of GIE in China’s provinces and cities, the regression coefficient of FDI_2_ on GIE is 0.5085 after exceeding the threshold value. It is significant at the 1% level, indicating that improving the quality of FDI substantially impacts GIE. The regression results of the third column show that when the level of knowledge accumulation in China’s provinces and cities is less than 9.1478, the regression coefficient of FDI_1_ on GIE is significantly negative, indicating that the increase in FDI_1_ can hinder the improvement of GIE. This may be due to the poor level of knowledge accumulation in China’s provinces and cities, the relatively weak ability of independent innovation, and the relatively low level of overall technology, so at this stage, the simple increase in the number and scale of FDI tends to form crowding-out effect and compliance cost effect on local enterprises in China’s provinces and cities, thus inhibiting the improvement of GIE. When the level of knowledge accumulation in China is more significant than 9.1478, the regression coefficient of FDI_1_ on GIE is significantly positive, indicating that the increase in FDI_1_can promote the GIE. This may be because with the improvement of knowledge accumulation in China’s provinces and cities, local innovation capability and technological level have made sufficient progress, so the simple quantitative expansion of FDI at this stage quickly forms spillover effects and innovation compensation effects on local enterprises in China’s provinces and cities, thus promoting the improvement of GIE. The regression results of the fourth column show that when the level of knowledge accumulation in China’s provinces and cities is less than 9.1478, the regression coefficient of FDI_2_ on GIE is significantly negative, indicating that the improvement of the quality of FDI can hinder the progress of GIE. When the level of knowledge accumulation in China’s provinces and cities is more significant than 9.1478, the regression coefficient of FDI_2_ on GIE is significantly positive, indicating that improving the quality of FDI can promote the GIE. In this regard, the above analysis fully justifies the assumptions of H2a and H2b.

**Fig 5:**
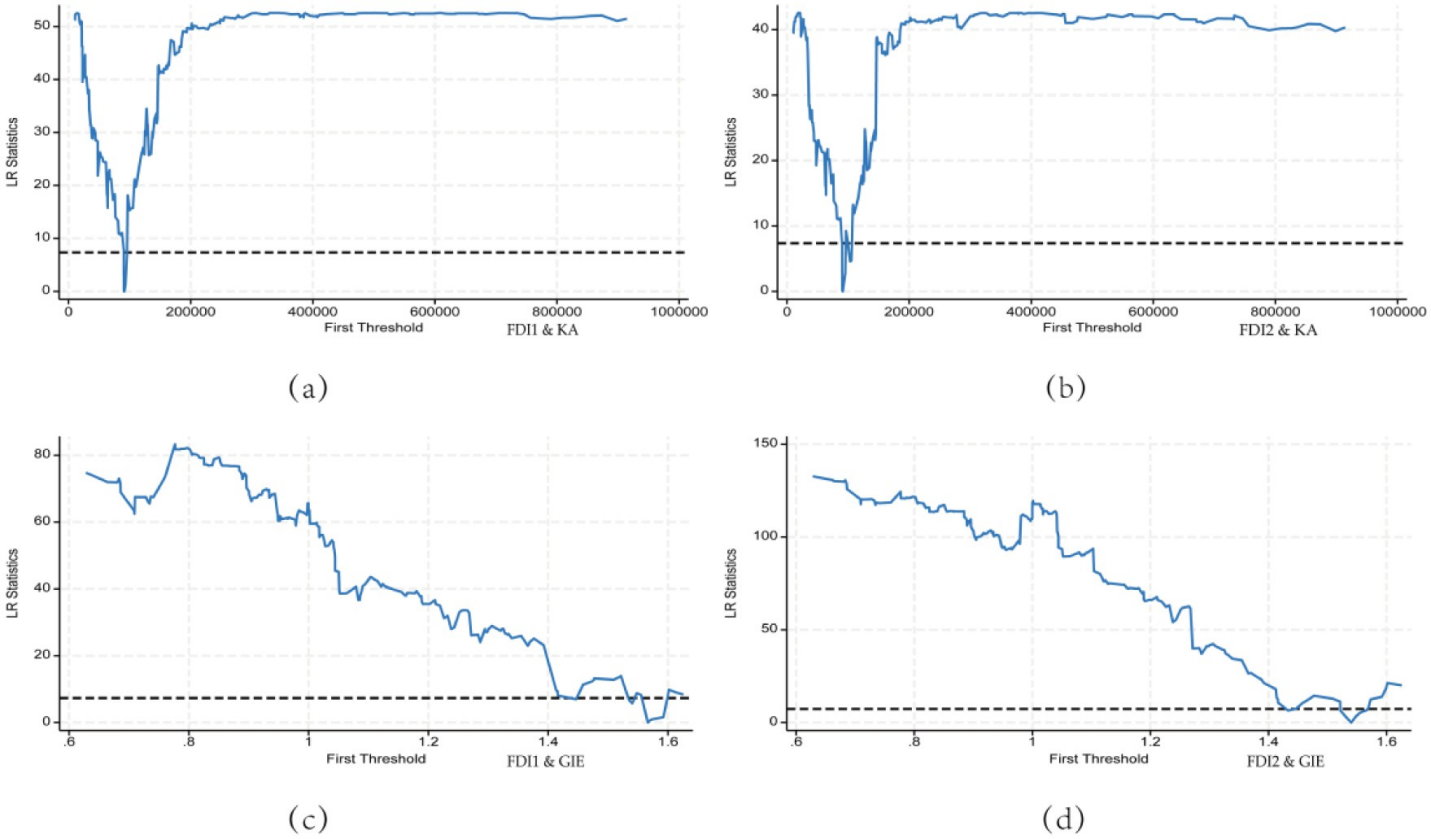
Threshold estimates for KA **(a b)** and GIE **(c d)**.

The threshold estimates of this paper are reported in Fig 5, where Fig (a) and (b) notify the threshold estimates for the threshold variable being KA, and Fig (c) and (d) report the threshold estimates for the threshold variable being GIE, respectively. As shown in Fig 5, the threshold point is the lowest in the LR graph.

## 7. Conclusion and policy recommendations

### 7.1 Conclusion

Based on panel data from 30 Chinese provinces spanning from 2011 to 2020, this study employs SDDF-GML model to evaluate GIE. Based on comprehensive benchmark regression analysis, mediation effect model, regional heterogeneity analysis, and threshold model, this paper establishes an empirical connection between the quantity and quality of FDI and GIE. The following objective conclusions have been obtained: Despite the presence of seven provinces evidencing a downward trend, China’s GIE has exhibited a general upward trend from 2011 to 2020. (2) The benchmark regression model demonstrates that FDI_1_ and FDI_2_ have a substantial impact on the GIE of Chinese provinces. This finding is consistent with those of previous studies [34, 37]. (3) The mediation effect model reveals that FDI_1_ and FDI_2_ can effectively improve the GIE of Chinese provinces by enhancing ER, with environmental regulation playing a significant role as mediator. (4) The examination of regional discrepancies indicates that FDI_1_ and FDI_2_ in districts with high education levels impede the progress of GIE to a lesser extent than in areas with low education levels. Within priority development areas, FDI_1_ and FDI_2_ hinder advancements in GIE. However, they possess the potential to mitigate or even promote the enhancement of GIE. In contrast, within key development areas, the quantity and quality of foreign direct investment encourage betterment of GIE. (5) The threshold model indicates that the effect of both FDI_1_ and FDI_2_ on the GIE in Chinese cities and provinces rises with the enhancement of GIE. When KA in Chinese provinces and cities falls below the threshold, the amplified FDI_1_ and FDI_2_ impede the growth of GIE. In contrast, when KA in Chinese provinces and cities surpasses the threshold, the heightened FDI_1_ and FDI_2_ supports the growth of GIE. This outcome corresponds with the findings outlined in [35].

### 7.2 Discussion

This paper confirms that both the quantity and quality of FDI can effectively promote GIE, which is consistent with previous studies [37, 40]. However, these previous studies also have some limitations, including the fact that the quantity and quality of FDI are not analyzed in the same framework. In our research, we fill this gap by providing new perspectives and insights for further research and policymaking in this area by comprehensively analyzing the impact of the quantity and quality of FDI on the efficiency of green innovation. Previous studies have rarely explored the mediating effect of environmental regulation in FDI and GIE, but this paper expands this research. Finally, when measuring the efficiency of green innovation, most of the previous studies have used SBM model [19] and EBM model [20]. However, compared with these models, the SDDF-GML model allows decision-makers to choose to reduce invalid inputs or increase the weight or direction of invalid outputs, thus increasing the flexibility and controllability of the model to a certain extent. This allows us to more accurately assess GIE and better understand the mechanisms by which FDI affects it.

The study’s results align partly with and partly complement previous research. It delves deeply into the mediating effect of environmental regulation levels and the heterogeneity of the primary functional areas to enrich the examination’s scope. It is noteworthy that two aspects need future enhancement: (1) data. This paper employs provincial data, which can be comprehensively analyzed at this level but makes carrying out more detailed analyses challenging and is incapable of reflecting the internal structure of the provincial level. To further validate the primary conclusions drawn in this paper, municipal data must be used. (2) Sample interval. The paper uses a sample interval from 2011–2020, as new data was not available and time limitations existed. However, incorporating new data could provide valuable insight for further testing.

### 7.3 Policy recommendations

Based on the analysis above, this paper recommends the following policies: (1) The spillover effect of FDI on green technology significantly promotes green technology innovation in China. Therefore, China should continue to attract green FDI and invest in domestic industries that are technology-intensive to promote the development of local green technology. (2) China should introduce high-quality FDI to encourage the green transformation of China’s industrial structure. China should establish a fair market competition environment and provide financial incentives to foreign enterprises with green technology spillover effects. This will promote the upgrading of industrial structure towards green initiatives. (3) In regions with high levels of education, it is imperative to implement market incentives that foster the green innovation impact of foreign direct investment (FDI). This may involve offering tax incentives, research and development (R&D) subsidies, and setting up bases for green technology innovation, with a view to drawing more FDI into green technology and, thus, promoting efficiency in green innovation. In regions with lower education levels, the government should persist in its efforts to enhance green innovation efficiency. This involves boosting investment in training talent for green technology, facilitating the conversion of scientific and technological progress, supporting local businesses and research institutions in innovating green technology, and delivering more convenient financial assistance and policy offerings. Governments can also increase their support for industries focused on green technology and direct more resources and investment towards green industries. This method can enhance green innovation efficiency and promote sustainable economic development at the local level. (4) Since the higher the level of knowledge accumulation, the more significant the contribution of FDI to the efficiency of green innovation, it is recommended that local governments increase the level of local knowledge accumulation. China could employ diverse methods, including tax incentives, encouraging businesses to increase investment in research and development, guiding various financial institutions to support independent innovation, improving policies to advance the conversion of scientific and technological achievements and the industrialisation of high and new technologies, to enhance local technological absorption capacity and knowledge. The assimilation of clean production technology through FDI will act as a driving force for enhancing the GIE in China’s provinces. To overcome the obstacles posed by FDI on the GIE, it is crucial to strengthen intellectual property protection and knowledge transformation, particularly when knowledge accumulation falls below the threshold. When knowledge accumulation surpasses a certain threshold, the government should sustain support for technological innovation and introduction to stimulate GIE growth via FDI. (5) As the impact of FDI on GIE elevates alongside improved GIE, local authorities should promote GIE growth from various angles. The literature confirms that the amelioration of GIE can be attained through government [31], industrial structure [32], and Internet development [21] can all contribute to the enhancement of GIE, and local governments can look for ways to promote the enhancement of GIE from these aspects.

## Supporting information

S1 File (DOCX)

S2 File (DO)

## Abbreviation

ER: Environmental Regulation
FDI: Foreign direct investment
GIE: Green innovation efficiency
KA: Level of knowledge accumulation
