# Supplementary material for: The impact of foreign direct investment on green innovation efficiency: Evidence from Chinese provinces
Source: PLoS One. 2024 Feb 14;19(2):e0298455. doi: 10.1371/journal.pone.0298455 (PMC10866484; doi:10.1371/journal.pone.0298455)
Supplement: S1 File — (DOCX) [file pone.0298455.s001.docx]

**Table 1** Significance test of Double threshold effect of quantity and quality of FDI

| TV | EV | Threshold | RSS | MSE | Fstat | Prob | Crit10 | Crit5 | Crit1 |
| --- | --- | --- | --- | --- | --- | --- | --- | --- | --- |
| GIE | FDI_1_ | Double | 10.0515 | 0.0347 | 40.54 | 0.0000 | 13.3415 | 15.9254 | 20.9802 |
| GIE | FDI_2_ | Double | 8.3857 | 0.0289 | 51.67 | 0.0000 | 11.1603 | 13.6299 | 19.8891 |
| KA | FDI_1_ | Double | 12.2650 | 0.0423 | 11.62 | 0.3633 | 18.2562 | 21.3144 | 31.5435 |
| KA | FDI_2_ | Double | 13.3735 | 0.0461 | -5.03 | 1.0000 | 18.8396 | 22.0466 | 29.7288 |

**Note**: TV is the threshold variable and EV is the explanatory variable

**Table 2** Double thresholds and confidence intervals for quantity and quality of FDI

| TV | EV | Threshold | Estimated threshold | 95% Confidence Interval | |
| --- | --- | --- | --- | --- | --- |
| GIE | FDI_1_ | Th-21 | 1.2381 | 1.1716 | 1.2431 |
| GIE | FDI_1_ | Th-22 | 1.5730 | 1.5622 | 1.5924 |
| GIE | FDI_2_ | Th-21 | 0.9553 | 0.9225 | 0.9645 |
| GIE | FDI_2_ | Th-22 | 1.5404 | 1.5282 | 1.5481 |
| KA | FDI_1_ | Th-21 | 3.9489 | 2.9693 | 4.0528 |
| KA | FDI_1_ | Th-22 | 9.1478 | 9.0365 | 9.3167 |
| KA | FDI_2_ | Th-21 | 6.3969 | 5.1995 | 6.4313 |
| KA | FDI_2_ | Th-22 | 13.7685 | 13.7387 | 14.0806 |

**Table 3** Parameter estimation results of FDI quantity and quality model

|  | (1) | (2) | (3) | (4) |
| --- | --- | --- | --- | --- |
| EV | GIE | GIE | GIE | GIE |
| TV | GIE | GIE | KA | KA |
| lnED | -0.5013^***^ | -0.3326^***^ | -0.1912 | -0.2612^*^ |
|  | (-3.85) | (-2.94) | (-1.35) | (-1.82) |
| lnFT | 0.0509 | 0.0166 | 0.0125 | 0.0077 |
|  | (0.77) | (0.28) | (0.17) | (0.10) |
| lnRD | -0.4671^***^ | -0.4309^***^ | -0.5887^***^ | -0.4094^***^ |
|  | (-3.73) | (-3.78) | (-4.13) | (-2.84) |
| lnMA | 0.3252^*^ | 0.1631 | 0.1451 | 0.1063 |
|  | (1.91) | (1.05) | (0.77) | (0.54) |
| lnURI | -1.4578^***^ | -1.4092^***^ | -1.3109^***^ | -1.2999^***^ |
|  | (-6.86) | (-7.24) | (-5.62) | (-5.29) |
| lnFD | 0.0676 | -0.0394 | 0.0454 | 0.1254 |
|  | (0.33) | (-0.21) | (0.20) | (0.54) |
| 0._cat#c.FDI1 | 0.0077 |  | -0.2389^***^ |  |
|  | (1.22) |  | (-6.49) |  |
| 1._cat#c.FDI1 | 0.0338^***^ |  | -0.1333^***^ |  |
|  | (4.79) |  | (-5.26) |  |
| 2._cat#c.FDI1 | 0.0897^***^ |  | 0.0145^**^ |  |
|  | (10.05) |  | (2.12) |  |
| 0._cat#c.FDI2 |  | -0.1530^***^ |  | -0.4475^***^ |
|  |  | (-3.90) |  | (-4.56) |
| 1._cat#c.FDI2 |  | 0.0582^**^ |  | -0.1107 |
|  |  | (2.15) |  | (-1.48) |
| 2._cat#c.FDI2 |  | 0.5199^***^ |  | 0.0894^***^ |
|  |  | (12.00) |  | (2.65) |
| _cons | 0.1798 | -1.0626 | -2.9212^*^ | -1.2654 |
|  | (0.13) | (-0.89) | (-1.97) | (-0.84) |
| *N* | 300 | 300 | 300 | 300 |
| adj. *R*^2^ | 0.4459 | 0.5377 | 0.3239 | 0.2628 |
